# Hypoperfusion in early-phase amyloid PET as a predictor of cognitive decline in Alzheimer's disease

**DOI:** 10.1007/s00259-025-07694-1

**Published:** 2026-03-02

**Authors:** A. Škorvagová, F. Ribaldi, J. Cerman, C. Wang, M. Scheffler, A. J Mendes, C. Wyss-Dominguez, V. Garibotto, G. Frisoni, R. Andel, C. Boccalini, J. Hort

**Affiliations:** 1 Memory Clinic, Department of Neurology, Charles University in Prague, 2Nd Faculty of Medicine and University Hospital Motol, Prague, Czech Republic; 2 Geneva Memory Center, Department of Rehabilitation and Geriatrics, Geneva University Hospitals, Geneva, Switzerland; 3 Laboratory of Neuroimaging of Aging (LANVIE), University of Geneva, Geneva, Switzerland; 4 Laboratory of Neuroimaging and Innovative Molecular Tracers (NIMTlab), Geneva University Neurocenter and Faculty of Medicine, University of Geneva, Geneva, Switzerland; 5 Division of Radiology, Geneva University Hospitals, Geneva, Switzerland; 6 Division of Nuclear Medicine and Molecular Imaging, Department of Medical Imaging, Geneva University Hospitals, Geneva, Switzerland; 7 Edson College of Nursing and Health Innovation, Phoenix , Arizona USA; 8 Genomic Molecular Diagnostics Laboratory, Diagnostic Department, Geneva University Hospitals, Geneva, Switzerland; 9 CIBM Center for Biomedical Imaging, Geneva, Switzerland

**Keywords:** Alzheimer disease, Amyloid PET, Cerebral perfusion, Early-phase imaging, Cognitive decline, Prognostic biomarker

## Abstract

**Purpose:**

Dual-phase amyloid PET enables concurrent assessment of amyloid burden and cerebral perfusion, providing both molecular and functional information within a single visit. While amyloid PET is well validated diagnostically, the prognostic value of early-phase perfusion remains less explored. We aimed to determine whether perfusion in Alzheimer’s disease (AD)–relevant regions is associated with longitudinal cognitive trajectories.

**Methods:**

We analyzed 218 participants from a memory-clinic cohort ranging from cognitively unimpaired (CU, n = 65) to mild cognitive impairment (MCI, n = 115) and dementia (n = 38). Based on visual read of late-phase amyloid PET, 123 participants were classified as amyloid-positive (Aβ+) and 93 as amyloid-negative (Aβ–). The groups differed significantly in age, education, and baseline MMSE (all p < 0.01), but not in sex distribution. Early-phase perfusion was quantified as standardized uptake value ratios (SUVr) within an AD meta–region of interest (posterior cingulate, angular, and temporal cortex) and analyzed using linear mixed-effects models adjusting for age, sex, education, APOE ε4 genotype, and white matter lesion burden.

**Results:**

Among Aβ+ participants, 11 were CU, 79 MCI, and 33 dementia; among Aβ– participants, 53 were CU, 35 MCI, and 5 dementia. Perfusion declined progressively across clinical stages (CU > MCI > dementia) and was positively correlated with baseline MMSE (r = 0.39, p < 0.001). In Aβ+ individuals, higher baseline perfusion predicted slower MMSE decline over time (interaction β = 5.85, p < 0.001), whereas no such association was observed in the Aβ– group. No significant association was observed in amyloid-negative participants. These effects remained significant after adjustment for vascular and genetic covariates and were independent of amyloid plaque burden.

**Conclusion:**

Perfusion derived from early-phase amyloid PET in AD-related regions is a reliable marker of cognitive status and progression in individuals with confirmed amyloid pathology. These findings highlight the added value of dual-phase PET as a practical, single-visit approach for combined diagnostic and prognostic evaluation, particularly where additional functional imaging is not feasible.

**Supplementary Information:**

The online version contains supplementary material available at 10.1007/s00259-025-07694-1.

## Introduction

Alzheimer’s disease (AD) pathology is identifiable through in vivo biomarkers. The ATN classification framework[1] categorizes individuals based on biomarkers of amyloid pathology (A), tau pathology (T), and neurodegeneration (N), and has been further refined to support biologically driven staging of the disease [2]. This model enables earlier diagnosis—often preceding symptom onset—and provides a standardized approach to disease classification.

For detecting Aβ plaques (A), both cerebrospinal fluid (CSF) Aβ42 and amyloid PET are considered gold-standard biomarkers. Amyloid PET plays a central role in research and clinical practice by providing a non-invasive, spatially resolved view of fibrillar amyloid deposition. Tracers such as [^1^⁸F]flutemetamol, [^1^⁸F]florbetapir, and [^1^⁸F]florbetaben are widely used to visualize amyloid burden in vivo. However, the amyloid PET signal plateaus early and shows limited correlation with cognitive symptoms, which reduces its value for disease staging[3, 4]. In contrast, FDG PET, which reflects cerebral glucose metabolism, shows the strongest association with current cognitive performance, exceeding that of both amyloid and tau PET [5–8]. Tau PET, on the other hand, captures the regional distribution of tau pathology and shows the best prognostic value for predicting future cognitive decline, as its patterns align closely with clinical phenotypes and neurodegeneration [9, 10].

A promising alternative is dual-phase amyloid PET, which captures both amyloid deposition and functional status in a single visit. The early phase, acquired within the first 5–10 min after tracer injection, reflects cerebral perfusion and serves as a surrogate for neuronal activity via the mechanism of neurovascular coupling [5, 11, 12]. Several validation studies have demonstrated that this early-phase signal closely parallels cerebral glucose metabolism measured by FDG-PET, providing comparable information on neuronal integrity [5, 11–14]. Neurovascular coupling refers to the tight physiological link between neuronal activity and local blood flow: increased synaptic activity triggers a proportional increase in perfusion [5]. In Alzheimer’s disease, however, amyloid-β accumulation disrupts this balance through endothelial dysfunction, vascular inflammation, and loss of vascular reactivity, leading to a state of neurovascular uncoupling [15]. These processes predominantly affect regions of the default mode network—such as the posterior cingulate and angular cortex—that are characterized by high baseline metabolic and synaptic activity and represent some of the earliest sites of amyloid deposition. Consequently, reductions in early-phase perfusion reflect not merely altered tracer delivery but genuine disturbances in neuronal and vascular function within these highly active, AD-vulnerable areas. The presence of amyloid pathology may further amplify these effects by promoting vascular dysregulation and synaptic stress in AD-relevant regions [15].

Because tau accumulation and related neuronal dysfunction emerge downstream of amyloid pathology, tau-PET studies consistently show the strongest correlation with cognitive decline [9, 10]. Early-phase amyloid PET perfusion may therefore capture this functional consequence of amyloid- and tau-related injury, bridging molecular (Aβ) and functional (N) components of the ATN framework within a single dual-phase acquisition.

A recent study by Völter et al. (2025) demonstrated that early-phase amyloid PET perfusion is associated with both current cognitive status and future cognitive decline in amyloid-positive individuals. In a focused cohort of 62 participants with confirmed amyloid pathology, they used a Braak stage–oriented ROI framework and showed that regional perfusion deficits—particularly in temporal and parietal areas—correlated with MMSE and CERAD-Plus scores at baseline and also predicted annual decline [16]. Building on this work, we aimed to extend these findings to a broader memory clinic population, including both amyloid-positive and amyloid-negative individuals, using a composite AD meta-ROI approach. Specifically, we investigated whether cerebral perfusion derived from dual-phase amyloid PET—originally developed for amyloid imaging in the late phase—would serve not only as a functional marker of neurodegeneration but also complement molecular information obtained from the late phase. 

## Methods

### Participants and clinical assessments

This retrospective cohort study included 218 individuals assessed at the Geneva Memory Center, a specialized tertiary clinic where patients undergo standardized clinical, neuropsychological, and imaging evaluations as part of their routine diagnostic work-up [17]. Enrollment and follow-up took place between 2016 and 2023. Participants were categorized into three clinical stages—cognitively unimpaired (CU), mild cognitive impairment (MCI), and dementia—based on established diagnostic criteria [18–20]. The CU group comprised both healthy volunteers and individuals with subjective cognitive decline.

Inclusion criteria for the present analyses were: (i) availability of a valid dual-phase amyloid PET scan with either [^1^⁸F]florbetapir or [^1^⁸F]flutemetamol, (ii) a structural T1-weighted MRI acquired within ± 1 year of the PET scan, and (iii) at least one follow-up MMSE assessment after baseline. Patients without follow-up cognitive data were excluded.

All participants underwent the imaging procedures as part of routine clinical care, and data were retrospectively analyzed. The study protocol was approved by the local ethics committee (CCER numbers: 2020–00403 and PB_2016-01346), and the research was conducted in accordance with the Declaration of Helsinki and International Conference on Harmonization Good Clinical Practice guidelines. All subjects or their legally authorized representatives had provided written informed consent for the use of their clinical and imaging data for research purposes.

### MRI acquisition and analysis

MRI was performed at Geneva University Hospitals using two different 3-T scanners depending on availability: the Magnetom Skyra (Siemens Healthineers, Erlangen, Germany) equipped with a 20- or 64-channel head coil, and the MR750w (GE Healthcare, Milwaukee, WI). Detailed acquisition parameters for both scanners are provided in Supplemental Material, Sect. 1. White matter lesions were visually rated using the age-related white matter change (ARWMC) scale [21].

### Amyloid PET acquisition and visual interpretation

Dual-phase amyloid PET was performed using Siemens Biograph PET/CT systems at the Division of Nuclear Medicine and Molecular Imaging, Geneva University Hospitals. Participants received either [^18^F]flutemetamol or [^18^F]florbetapir. The early-phase scan began immediately after tracer injection (5 min for florbetapir, 10 min for flutemetamol), according to manufacturer recommendations; the late-phase scan followed standardized timing protocols (approximately 50 min post-injection for florbetapir and 90 min for flutemetamol). Amyloid status (Aβ +/Aβ −) was determined by visual read of late-phase PET by an experienced nuclear medicine physician in accordance with EMA-approved criteria. Centiloid values were computed and used in complementary analyses (see Statistical Analysis). Two participants with borderline reads were excluded from stratified (Aβ +/Aβ −) analyses but retained in overall models where appropriate. Full acquisition protocols are detailed in Supplemental Materials, Section 2.

### Early-phase PET processing and perfusion quantification

All PET and MRI data were preprocessed using Statistical Parametric Mapping (SPM12) in MATLAB R2018b. Steps included co-registration, spatial normalization, and region-based signal extraction (see Supplemental Materials, Section 3).

Early-phase PET images were used to estimate cerebral perfusion via standardized uptake value ratios (SUVr), normalized to the mean signal in a pons–cerebellar vermis composite. This reference region was selected following the ADNI implementation by Landau et al. (2011) and subsequent dual-phase PET validations [5, 6], as it shows minimal susceptibility to Alzheimer’s-related perfusion or amyloid changes and avoids potential spill-in from nonspecific uptake in the cerebellar cortex.

For target quantification, we used a composite AD meta-ROI originally defined by Landau et al. (2011) for FDG-PET studies. This ROI comprises the bilateral angular gyri, posterior cingulate cortex, and left middle/inferior temporal gyrus, derived empirically from AD-related hypometabolic regions identified in FDG-PET studies. The meta-ROI captures temporoparietal regions most sensitive to AD-related hypometabolism and has been widely adopted for both FDG- and early-phase amyloid-PET analyses [5, 22].

ROI extraction was performed using the AAL3 atlas, and mean SUV values were extracted voxelwise. For each participant, volume-weighted averages were computed within the target and reference masks to account for regional size differences, and a composite perfusion SUVr was then calculated.

### Statistical analysis

Demographic and clinical characteristics were summarized for the full cohort (n = 218), including age, sex, education, clinical stage, MMSE scores, and amyloid status. Baseline amyloid burden (Centiloid values) and APOE ε4 carrier status were also summarized in Table 1 to provide a comprehensive overview of molecular and genetic characteristics, while detailed subset summaries are presented in Supplementary Tables S1 and S2. Group counts and proportions for clinical stages and amyloid subgroups are reported in the Results section.

**Table 1:** Baseline demographics and key covariates

Baseline demographics and key covariates			
	All	AB +	AB-
N	218	123	93
Age (years)	70.9 ± 7.4	72.8 ± 6.4	68.6 ± 7.7
Female (%)	43.6%	44.74%	41.9%
Education (years)	13.7 ± 4.1	13.2 ± 4.2	14.4 ± 3.9
MMSE (baseline)	25.7 ± 4.1	24.4 ± 4.5	27.5 ± 2.7
CU/MCI/DEM (n)	65/115/38	11/79/33	53/35/5
Centiloid (mean ± SD)	43.1 ± 45.6	76.1 ± 32.1	−0.4 ± 12.6
APOE ε4 availability (n)	140	61	77
0 ε4 alleles (n)	85	23	61
1 ε4 alleles (n)	47	30	16
2 ε4 alleles (n)	8	8	0
ARWMC availability (n)	155	99	56
ARWMC (mean ± SD)	6.1 ± 4.6	5.7 ± 4.1	6.7 ± 5.5

Values are mean ± SD unless stated otherwise. All values include Aβ + and Aβ– participants; two cases with borderline visual reads were excluded from stratified analyses but retained in the total sample. Cases with missing PET data are excluded. APOE and ARWMC rows show availability (n); their summaries are computed on available data only. ARWMC = Age-Related White Matter Changes, visually rated on FLAIR MRI using the *Wahlund *et al*., 2001* scale (range 0–30).

Analyses were conducted on available data for each variable. Because APOE genotype and ARWMC scores were not available for all participants, missingness mechanisms were assessed using logistic regression. Missingness was not completely at random; therefore, multiple imputation was deemed inappropriate, and a complete-case approach was used to avoid introducing bias.

Associations between early-phase perfusion and cognitive status at baseline were assessed using Pearson correlation and linear regression, with MMSE as the dependent variable. Group differences in perfusion across diagnostic stages (CU, MCI, DEM) were tested using one-way ANOVA or Kruskal–Wallis tests, followed by Tukey’s HSD or ANCOVA adjusting for age and sex, as appropriate. In addition, the association between perfusion SUVr and Centiloid values was examined using Spearman correlation to explore the correspondence between perfusion and amyloid burden. All cross-sectional analyses were also repeated within Aβ + and Aβ– subgroups to explore potential differences based on amyloid status.

Longitudinal changes in MMSE were modeled using linear mixed-effects models (LME) with random intercepts and slopes (time | subject) to capture individual variability in baseline cognition and rate of decline. Continuous predictors were mean-centered for interpretability. Because APOE and ARWMC data were available only for subsets, models were built sequentially (Models 1–3) with incremental covariate adjustment rather than a single saturated model. This approach maximized sample size for each model while minimizing bias related to variable-specific missingness. Exact *lmer()* model formulas and full parameter estimates are provided in Supplementary Section 5, where Holm–Bonferroni correction was applied separately to main and interaction effects.

To directly compare functional (perfusion) and molecular (amyloid) predictors, a complementary LME model was fitted in which Centiloid values replaced perfusion SUVr as the primary continuous predictor of MMSE, using identical covariates and random-effects structure. An additional joint model included both perfusion SUVr and Centiloid values, along with their interactions with time, to evaluate their independent and additive predictive effects on longitudinal cognitive trajectories.

To explore the biological relationship between functional and molecular PET measures, the association between early-phase perfusion SUVr and global amyloid burden (Centiloid values) was further examined using both Spearman correlation and linear regression. In addition, a logistic regression and ROC analysis were performed to evaluate the ability of perfusion SUVr to classify amyloid positivity defined by a Centiloid threshold of ≥ 20.

Fixed effects across all models included baseline perfusion (or Centiloid), time, their interaction, age, sex, and education. Extended models added amyloid status, APOE ε4 status, and ARWMC as covariates. Analyses were repeated separately in amyloid-positive and amyloid-negative subgroups to examine potential differences in effect direction and magnitude. Sensitivity analyses stratified models by PET tracer ([^1^⁸F]florbetapir vs [^1^⁸F]flutemetamol) to verify that perfusion estimates were not tracer-dependent.

Then we tested an exploratory mixed-effects model including both perfusion SUVr (Composite) and Centiloid as continuous variables, as well as their interaction with time, to examine whether the relationship between perfusion and cognitive change differed across levels of amyloid burden.

Finally, to evaluate prognostic value, a Cox proportional-hazards model was fitted to predict time to cognitive decline, defined as a ≥ 3-point decrease in MMSE at any follow-up relative to baseline, regardless of subsequent recovery. Covariates included baseline perfusion, Centiloid, age, sex, and education.

All analyses were conducted in R (version 4.3.2) using the *lme4*, *lmerTest*, *survival*, *ggplot2*, and *ggeffects* packages. Two-tailed p < 0.05 was considered statistically significant.

## Results

### Demographic and clinical characteristics

The study included 218 participants (65 CU, 115 MCI, 38 DEM). Based on visual read of late-phase amyloid PET, 123 were classified as amyloid-positive (Aβ +), 93 as amyloid-negative (Aβ–), and 2 as borderline. The borderline cases were excluded from stratified analyses but retained in models including the full cohort. Among Aβ+ participants, 11 were CU, 79 had MCI, and 33 had dementia. In the Aβ– group, 53 were CU, 35 had MCI, and 5 had dementia. Aβ+ participants were older and had lower MMSE scores than Aβ– participants (p < 0.001).

Mean age of the total sample was 70.9 ± 7.4 years, and 43.6% were women. Average education was 13.7 ± 4.1 years. Baseline MMSE was 25.7 ± 4.1, with lower scores in Aβ + (24.4 ± 4.5) than in Aβ– participants (27.5 ± 2.7). Centiloid values differed markedly between groups (Aβ + : 76.1 ± 32.1; Aβ–: –0.4 ± 12.6), consistent with amyloid status classification.

APOE genotype data were available for 140 participants (64%), of whom 47 carried one ε4 allele and 8 carried two. ARWMC ratings were available for 155 participants (71%) and averaged 6.1 ± 4.6 on the Wahlund scale (range 0–30). Compared with Aβ– individuals, Aβ+ participants were older, had fewer years of education, and showed lower MMSE and higher Centiloid values. Full demographic and imaging summaries are presented in Table 1.

### Perfusion differences across clinical groups

Across the full cohort (CU: n = 65, MCI: n = 115, DEM: n = 38), descriptive statistics showed distinct mean perfusion values in each group: CU = 1.28 ± 0.13, MCI = 1.24 ± 0.11, and DEM = 1.13 ± 0.12. A one-way ANOVA revealed a significant overall difference in perfusion across clinical stages [F(2, 214) = 21.09, p < 0.001]. Post-hoc comparisons using Tukey’s HSD test indicated that participants with dementia had significantly lower perfusion compared with both CU (mean difference = –0.15, 95% CI [–0.21, –0.10], p < 0.001) and MCI (mean difference = –0.11, 95% CI [–0.16, –0.06], p < 0.001). Additionally, perfusion in MCI was significantly lower than in CU (mean difference = –0.05, 95% CI [–0.09, –0.002], p = 0.036).

To assess whether this pattern differed by amyloid status, we repeated the analysis separately for Aβ– (n = 93) and Aβ+ (n = 123) subgroups. Among Aβ– participants, perfusion differed significantly across clinical stages [F(2, 90) = 5.87, p = 0.004]. Post-hoc tests showed that participants with dementia had significantly lower perfusion compared with both CU (mean difference = –0.19, 95% CI [–0.32, –0.06], p = 0.003) and MCI (mean difference = –0.18, 95% CI [–0.31, –0.05], p = 0.005), with no significant difference between CU and MCI.

In the Aβ + group, perfusion also varied significantly by clinical stage [F(2, 120) = 9.63, p < 0.001]. Participants with dementia had lower perfusion compared with CU (mean difference = –0.16, 95% CI [–0.25, –0.06], p = 0.001) and MCI (mean difference = –0.09, 95% CI [–0.14, –0.03], p = 0.001). Again, no significant difference was observed between CU and MCI in this subgroup.

**Fig. 1:**
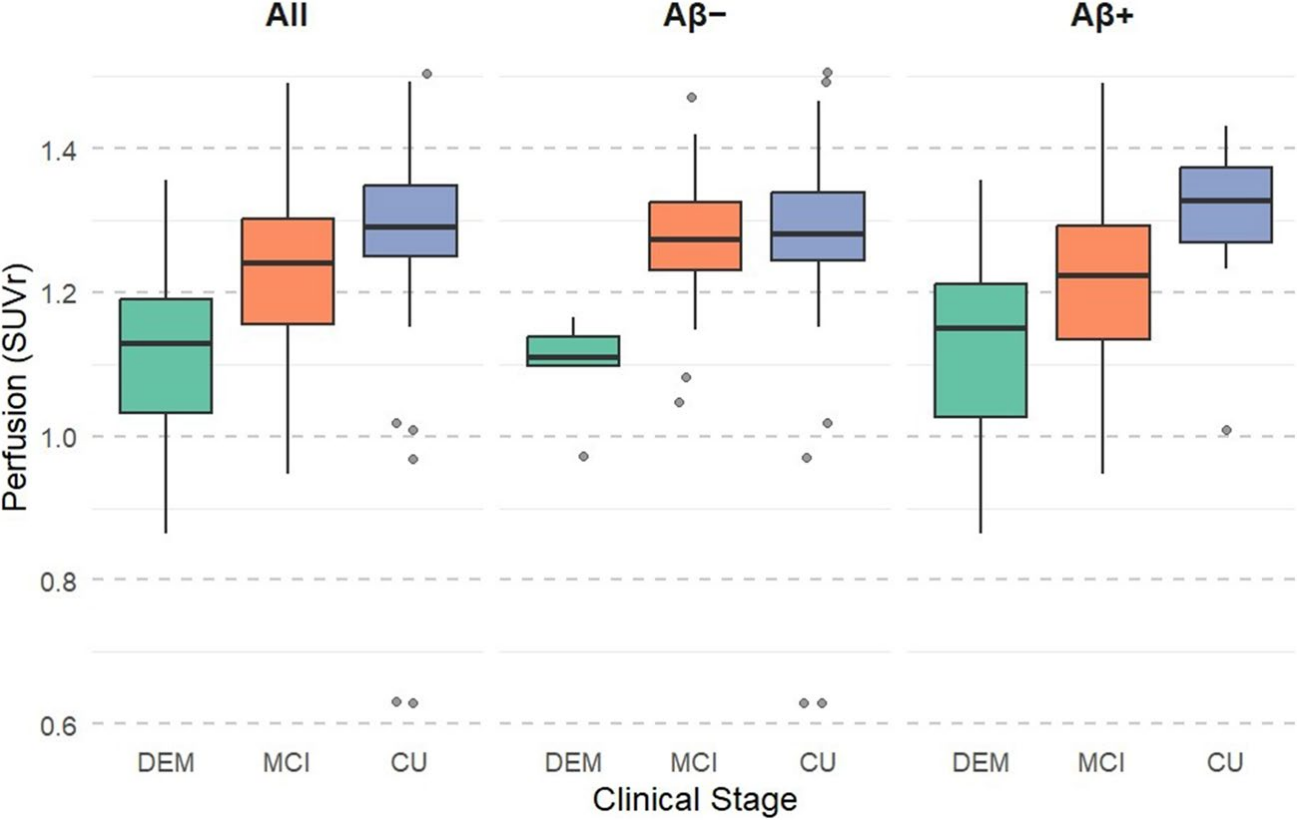
Perfusion by clinical stage and amyloid status. Boxplots show early-phase PET perfusion values (meta-ROI SUVr) across diagnostic groups:CU, MCI, DEM. The left panel displays values for the full sample, while the middle and right panels show results stratified by amyloid PET status (Aβ − and Aβ +, respectively). Perfusion was lowest in the dementia group and progressively higher in MCI and CU, consistent with a decline in cerebral perfusion across the clinical continuum of cognitive impairment. This pattern was more pronounced in Aβ+ individuals

**Fig. 2:**
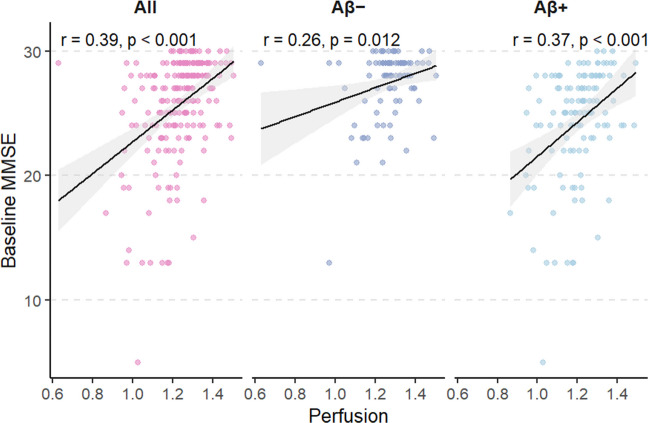
Baseline relationship between perfusion and MMSE across amyloid subgroups. Scatterplots show the positive association between early-phase PET perfusion values and baseline MMSE in the full cohort (left), Aβ- (middle), and Aβ + (right) groups. Pearson correlation coefficients (r) and p-values are displayed in each panel. The association was strongest in the Aβ + group (r = 0.37, p < 0.001), but remained significant in both groups

**Fig. 3:**
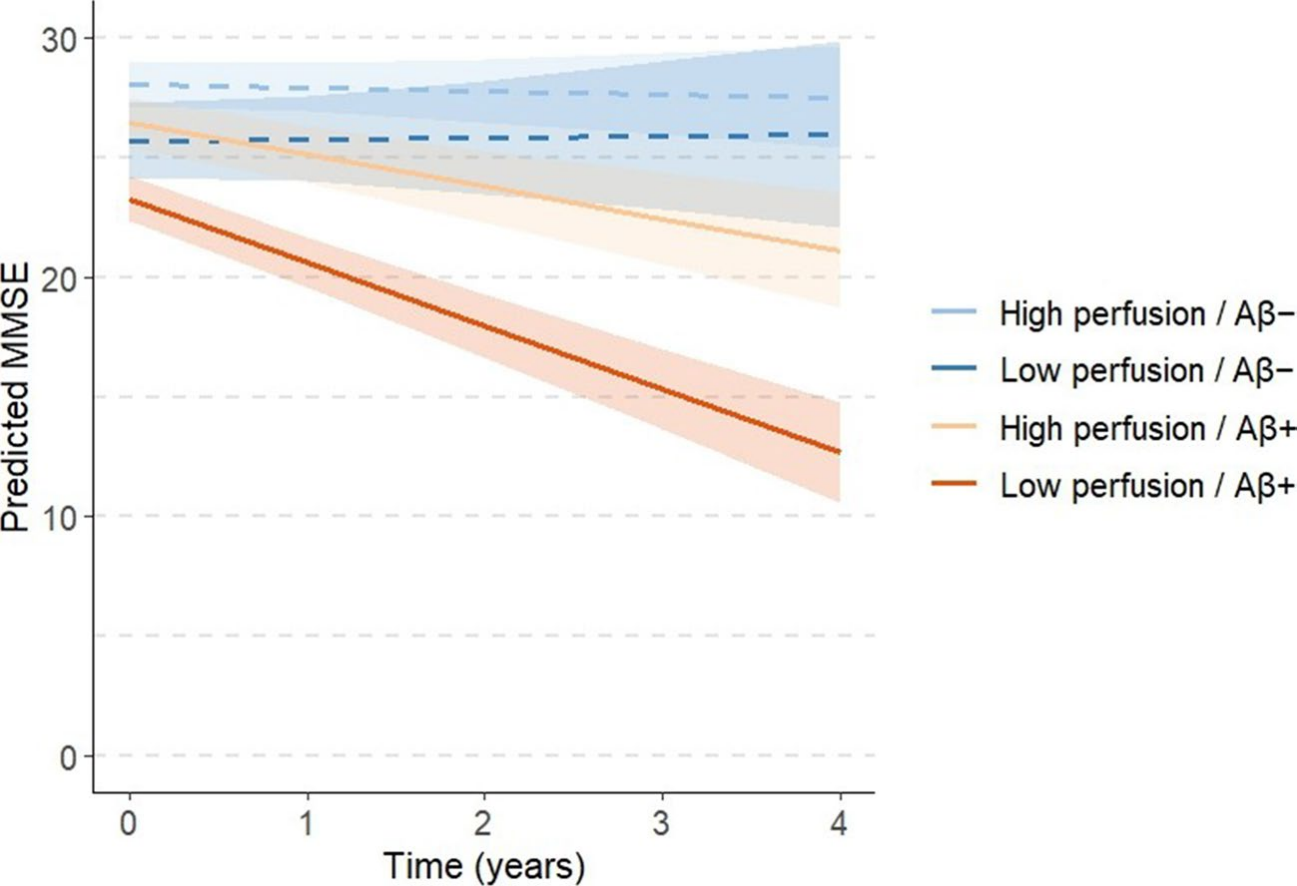
Predicted MMSE trajectories over time based on perfusion and amyloid status. Participants were stratified into low and high perfusion groups using a data-driven cut-off (Youden index) based on early-phase PET perfusion values. Cognitive trajectories (MMSE) were estimated using linear mixed-effects models adjusted for age, gender, and years of education. Solid lines represent Aβ+ individuals, and dashed lines represent Aβ− individuals. Shaded areas represent 95% confidence intervals. Individuals with higher perfusion showed slower cognitive decline, but this effect was evident primarily in the Aβ + group

In addition to within-group analyses, we compared overall perfusion between Aβ + and Aβ– participants across the full cohort. Aβ+ individuals showed significantly lower mean perfusion (1.21 ± 0.12) compared with Aβ– individuals (1.27 ± 0.13), t(216) = 3.43, p = 0.0007. Detailed group means and ANOVA results are summarized in Supplementary Table S4. Figs. 1, 2 and 3

### Perfusion and cognition

The relationship between perfusion, as measured by early-phase amyloid PET in AD-related meta-ROI regions, and cognitive function, assessed using the MMSE, was examined cross-sectionally using correlation analyses. Across the entire cohort, a significant positive Pearson correlation was observed (r = 0.39, p < 0.001), indicating that higher perfusion levels were associated with better cognitive performance.

This relationship was further supported by an exploratory linear regression (β = 12.83, SE = 2.04, p < 0.001), which explained a modest proportion of variance (R^2^ = 0.16), consistent with a moderate linear association.

When analyses were repeated within amyloid subgroups, the association was weaker in Aβ– participants (r = 0.26, p = 0.012) and stronger in Aβ+ participants (r = 0.37, p < 0.001). As a comparison, higher amyloid burden (Centiloid) was inversely related to baseline MMSE (r = –0.32, 95% CI [–0.43, –0.21], p < 0.001; see Supplementary Section 5).

In addition to cognitive performance, we also examined the biological relationship between perfusion and amyloid burden. Early-phase perfusion SUVr showed a modest but significant inverse association with Centiloid values (Spearman ρ = –0.26, p = 0.0001; Pearson r = –0.23, p = 0.0006), consistent with partially independent contributions of amyloid burden and perfusion. Linear regression indicated a negative slope between Centiloid and perfusion (β = –0.0006, p = 0.0006; Adj. R^2^ = 0.05), while classification analysis yielded AUC = 0.68 for distinguishing amyloid-positive cases (≥ 20 CL). These findings are illustrated in Supplementary Figure S1 and summarized in Supplementary Tables S13–S14.

### Longitudinal associations between perfusion and MMSE

To assess how baseline perfusion relates to cognitive trajectories, longitudinal linear mixed-effects (LME) models were fitted using repeated MMSE scores as the dependent variable. Across the full cohort (Model 1a; 644 observations from 218 participants), higher baseline perfusion was associated with both higher initial MMSE (β = 10.24, p < 0.001) and a slower rate of decline over time (perfusion × time interaction = 4.54, p < 0.001). These effects remained significant after Holm–Bonferroni correction (Tables S5–S6).

When analyses were stratified by amyloid status, the association between perfusion and cognition was mainly evident among amyloid-positive individuals. In the Aβ + subgroup (Model 1c; 377 observations from 123 participants), higher perfusion predicted both better baseline cognition (β = 13.04, p < 0.001) and slower decline (perfusion × time interaction = 5.85, p < 0.001), whereas in the Aβ– subgroup (Model 1b; 258 observations from 93 participants), perfusion related only to baseline MMSE (β = 4.88, p = 0.03) and not to change over time (perfusion × time interaction = –0.48, p = 0.61; Tables S7–S8). Exact R syntax used for Models 1–3 is provided in Table S17.

Including additional biological covariates did not materially alter these associations.In Model 2, which incorporated MRI-based white matter lesion burden (ARWMC), perfusion remained strongly associated with both higher baseline MMSE (β = 13.96, 95% CI [9.25, 18.67], p < 0.001) and slower decline (perfusion × time interaction: 6.17, 95% CI [3.14, 9.20], p < 0.001), whereas ARWMC showed no significant association with cognition (β = 0.01, 95% CI [–0.12, 0.13], p = 0.901; Table S9).

In Model 3, which added APOE genotype, perfusion continued to predict both higher baseline cognition (β = 4.60, 95% CI [0.87, 8.34], p = 0.016) and slower decline over time (perfusion × time interaction = 3.55, 95% CI [1.03, 6.08], p = 0.006). APOE ε4 homozygosity was associated with lower MMSE scores (β = –2.51, 95% CI [–4.55, –0.46], p = 0.017), whereas heterozygous ε4 carriers did not differ significantly from non-carriers. In this smaller subset, the effect of education did not reach significance, likely reflecting reduced statistical power rather than a true absence of association (Table S10).

To test whether the effect of perfusion on cognition differed by amyloid status, we fit an interaction model including perfusion, amyloid positivity, and their interaction term, along with time, age, sex, and education (Table S11; 644 observations from 218 participants). Perfusion was significantly associated with higher baseline MMSE (β = 6.06, p = 0.046), and amyloid positivity with lower MMSE scores (β = –2.11, p < 0.001). The three-way interaction between perfusion, amyloid status, and time was significant (β = 7.16, p = 0.002), indicating that higher perfusion predicted slower cognitive decline over time, particularly in Aβ+ individuals. Although the two-way perfusion × amyloid term did not reach significance (β = 7.45, p = 0.062), the direction was consistent with the subgroup-specific models, supporting a stronger perfusion–cognition association in the amyloid-positive group.

Perfusion and amyloid burden were only modestly correlated (Spearman ρ = –0.26, p = 0.0001; Table S12), indicating partially independent biological contributions. A logistic model using perfusion SUVr to classify amyloid positivity (≥ 20 Centiloids, visual read–consistent threshold) achieved an AUC = 0.68, suggesting limited discriminative accuracy (Table S15). When Centiloid values replaced perfusion as the continuous predictor (Table S13), higher amyloid burden predicted both lower baseline MMSE and faster decline (Centiloid × time: β = –0.02 [–0.03, –0.01], p < 0.001). The Centiloid model showed a slightly better overall fit (AIC = 3410 vs. 3431; ΔAIC ≈ –20; Table S14), yet perfusion exhibited a stronger standardized association with MMSE slope, consistent with complementary rather than redundant predictive value.

In an exploratory extension of this model including continuous Centiloid values and their interaction with perfusion, both perfusion and Centiloid independently predicted baseline MMSE (β = 10.1, p = 0.019; β = –0.016, p = 0.015, respectively). A significant three-way interaction (perfusion × Centiloid × time: β = 0.072, p = 0.041) indicated that the protective effect of higher perfusion on cognitive decline was strongest in individuals with greater amyloid burden (Table S16).

InFinally, Cox proportional-hazards analysis indicated that higher baseline perfusion was associated with a markedly reduced risk of cognitive decline (≥ 3-point MMSE decrease; HR = 0.10 [0.02–0.43], p = 0.0018), while higher Centiloid values predicted increased risk (HR = 1.013 [1.009–1.018], p < 0.0001). Education was protective (HR = 0.90, p < 0.001) (Table 2).

**Table 2:** Linear Mixed–Effects Models (1a-3): Fixed Effects Summary

Predictor		95% CI	p-value
Model 1a (Full sample)-n = 218 participants 644 observations			
Age	0.01	[−0.06, 0.08]	0.810
Education	0.27	[0.15, 0.39]	< 0.001
Gender (female)	−0.73	[−1.72, 0.26]	0.147
Perfusion	12.04	[8.11, 15.96]	< 0.001
Perfusion x time	4.46	[2.14, 6.79]	< 0.001
Model 1b (Aβ-)-n = 93 participants 258 observations			
Age	−0.01	[−0.08, 0.06]	0.863
Education	0.25	[0.12, 0.39]	< 0.001
Gender (female)	−1.24	[−2.33, −0.16]	0.025
Perfusion	4.88	[0.44, 9.31]	0.031
Perfusion x time	−0.48	[−2.32, 1.36]	0.610
Model 1c (Aβ +)-n = 123 participants 379 observations			
Age	0.07	[−0.05, 0.18]	0.244
Education	0.28	[0.11, 0.45]	0.002
Gender (female)	–0.45	[−1.89, 0.98]	0.534
Perfusion	13.08	[7.21, 18.95]	< 0.001
Perfusion x time	5.84	[2.48, 9.21]	< 0.001
Model 2 (+ ARWMC)-n = 155 participants 480 observations			
Age	0.03	[−0.06, 0.12]	0.530
Education	0.31	[0.17, 0.44]	< 0.001
Gender (female)	−0.97	[−2.10, 0.17]	0.094
Perfusion	13.96	[9.25, 18.67]	< 0.001
Perfusion x time	6.17	[3.14, 9.20]	< 0.001
White matter changes (ARWMC)	0.01	[−0.12, 0.13]	0.901
Model 3 (+ APOE)-n = 140 participants 410 observations			
Age	−0.06	[−0.12, 0.00]	0.058
Education	0.08	[−0.04, 0.20]	0.209
Gender (female)	0.00	[−0.92, 0.92]	0.996
Perfusion	4.60	[0.87, 8.34]	0.016
Perfusion x time	3.55	[1.03, 6.08]	0.006
All models include random intercepts and slopes (time | ID). Shown predictors are common across models: full categorical contrasts for APOE are reported in Table S10. Estimates and p-values via ImerTest (Satterthwaite)			

Summary of fixed effects from linear mixed-effects models (Models 1–3) examining the association between baseline perfusion and longitudinal MMSE performance. Shown are β estimates, 95% confidence intervals (CI), and p-values for selected predictors, including baseline perfusion, the interaction between perfusion and time, and APOE genotype. All models included random intercepts and slopes (time | subject), and were adjusted for age, sex, and years of education. Model 1a includes the full sample; Models 1b and 1c are stratified by amyloid PET status (Aβ − and Aβ +, respectively). Model 2 additionally adjusts for white matter lesion burden.Model 3 includes APOE ε4 genotype, coded as the number of ε4 alleles (0, 1, or 2). ARWMC—age-related white matter changes scale.

## Discussion

This study examined whether early-phase amyloid PET–derived perfusion predicts longitudinal cognitive decline independently of traditional risk factors, including amyloid plaque burden, APOE ε4 genotype, and white matter lesion load. Our findings demonstrate that perfusion in Alzheimer’s disease–sensitive regions is a robust cross-sectional correlate of global cognition and a significant predictor of subsequent cognitive decline, but only among individuals with confirmed amyloid pathology. These results underscore the clinical relevance of early-phase amyloid PET as a practical dual biomarker integrating molecular (Aβ) and functional (perfusion) information within a single session [14, 23].

### Perfusion differences across clinical stages

Perfusion values extracted from AD-sensitive meta-ROIs declined progressively along the spectrum of cognitive impairment, following the expected clinical continuum from cognitively unimpaired to dementia. This gradient mirrors functional deterioration typically observed in FDG-PET studies and supports the interpretation of early-phase amyloid PET as a proxy of neuronal integrity [7, 8, 24]. In the full sample, perfusion differed significantly across all diagnostic stages, with a stepwise reduction from CU to MCI and dementia. However, after stratification by amyloid status, differences between CU and MCI were no longer significant, which may reflect reduced statistical power in smaller subgroups and substantial heterogeneity in amyloid burden and underlying pathology within the MCI category [24, 25]. Importantly, within both amyloid-positive and amyloid-negative groups, perfusion in the dementia stage remained markedly lower than in CU and MCI participants, indicating that advanced-stage hypoperfusion represents a common downstream feature of neurodegeneration, independent of amyloid status. This pattern aligns with previous perfusion imaging studies showing a continuous decline in cerebral blood flow along the Alzheimer’s disease continuum [13].

### Perfusion and baseline cognition

We observed a moderate but significant positive correlation between perfusion and MMSE scores at baseline, indicating that higher perfusion in AD-vulnerable regions corresponds to better global cognitive performance. This association was present in both amyloid-negative and amyloid-positive participants but was notably stronger in the latter. Because the meta-ROI used in this study encompasses temporoparietal and posterior cingulate regions most affected by Alzheimer’s pathology, this pattern likely reflects the region-specific sensitivity of the perfusion signal to AD-related functional changes. These results align with previous findings showing that early-phase amyloid PET provides a reliable proxy for cerebral perfusion that parallels glucose metabolism and cognitive performance [11, 13].

Although perfusion and amyloid deposition were modestly correlated (ρ = –0.26), this relationship explained only a small portion of the variance, suggesting that early-phase perfusion and amyloid burden capture complementary aspects of Alzheimer’s disease pathophysiology. The limited discriminative ability of perfusion SUVr for amyloid positivity (AUC = 0.68) is consistent with previous work showing only moderate coupling between early-phase perfusion and late-phase amyloid signal [11, 13]. This supports the interpretation that perfusion provides functionally distinct information related to neuronal and vascular integrity rather than direct tracer binding.

The absence of a significant association in the Aβ − subgroup should be interpreted with caution. Early-phase perfusion derived from amyloid tracers provides physiologically valid information but may exhibit a lower signal-to-noise ratio in individuals without cortical amyloid binding, particularly when analyses focus on AD-specific temporoparietal regions [5, 14]. While this lower sensitivity likely contributes to the weaker associations observed in Aβ − participants, it does not fully explain the absence of a longitudinal effect.

In fact, the lack of association in the Aβ − group may reflect distinct underlying pathophysiological mechanisms rather than mere methodological limitations. Hypoperfusion in Aβ− individuals is frequently linked to cerebrovascular pathology or non-AD proteinopathies such as TDP-43 or frontotemporal degeneration, which may follow trajectories that diverge from the Alzheimer’s continuum. Consistent with this interpretation, the significant perfusion × amyloid × time interaction (β = 7.16, p = 0.002) indicates that the relationship between perfusion and cognitive decline differs systematically across amyloid strata, supporting a biological rather than noise-driven origin of the effect. Future work should extend these analyses to region-specific and voxel-wise approaches to disentangle AD-specific perfusion deficits from those associated with other etiologies.

### Perfusion predicts longitudinal cognitive decline

In longitudinal analyses, higher baseline perfusion predicted slower MMSE decline among amyloid-positive participants, whereas no significant association was observed in the amyloid-negative group. Although the perfusion × amyloid interaction did not reach statistical significance, the direction and magnitude of effects were consistent across stratified models, indicating that perfusion serves as a functional marker of disease progression. These associations remained robust after adjustment for age, sex, education, white matter lesion burden, and APOE genotype, suggesting that perfusion provides prognostic information beyond traditional demographic and vascular factors.

APOE status itself was not an independent predictor, although ε4 homozygotes showed a non-significant trend toward lower MMSE scores. A joint model including both perfusion and amyloid burden further demonstrated their additive predictive value: perfusion and Centiloid values independently predicted the rate of cognitive decline, confirming that both functional and molecular biomarkers contribute distinct yet complementary information on disease progression.

Despite a modest inverse correlation between perfusion SUVr and Centiloid burden (ρ = –0.26), both measures were independently related to cognitive change. Exploratory interaction analyses further supported this interpretation: the significant perfusion × Centiloid × time interaction (β = 0.072, p = 0.041) indicated that the protective effect of higher perfusion on cognitive decline was strongest in individuals with greater amyloid burden. This finding supports a synergistic interplay between vascular–neuronal function and amyloid pathology, consistent with the view that these processes jointly modulate disease progression.

Although the Centiloid model achieved a slightly better overall fit, perfusion showed a stronger standardized association with MMSE slope, underscoring its closer relationship to real-time neuronal dysfunction. Together, these results suggest that amyloid accumulation represents an early and relatively static process, whereas perfusion decline constitutes a downstream and more dynamic indicator of neuronal and vascular compromise. In survival analyses, higher baseline perfusion was associated with a substantially lower hazard of clinically meaningful decline, highlighting its potential utility for patient-level risk stratification.

Mechanistically, early-phase amyloid PET reflects regional cerebral blood flow tightly coupled to neuronal activity [11, 13]. In Alzheimer’s disease, amyloid-related vascular and synaptic dysfunction disrupt this coupling [15], creating a functional cascade from amyloid (A) to tau-mediated neuronal injury (T/N) and cognitive decline (C) within the ATN framework [1, 10, 26]. Although tau-PET data were not available in the present study, prior work has demonstrated a strong spatial overlap between tau accumulation and functional impairment measured by FDG- or early-phase amyloid PET [9, 10, 26]. These converging findings suggest that perfusion captures downstream neuronal vulnerability associated with tau pathology. Consistent with this interpretation, perfusion contributed prognostic information beyond amyloid burden and other risk factors, underscoring its complementary value as a dynamic functional marker within the ATN model.

### Comparison with previous studies

Our findings extend those of [16], who reported that early-phase amyloid PET hypoperfusion predicted subsequent cognitive decline in a smaller, amyloid-positive cohort using Braak stage–based regions of interest. By including both amyloid-positive and amyloid-negative individuals and applying a meta-analytically defined AD ROI, our study replicates and broadens these results in a clinically heterogeneous memory-clinic population. Moreover, by using linear mixed-effects modeling across repeated MMSE assessments, we were able to capture individual rates of change over several years, providing stronger evidence for the prognostic value of perfusion-derived measures.

Beyond Völter’s study, multiple investigations have shown that the early phase of amyloid PET reliably reflects cerebral perfusion and can serve as a surrogate for FDG-PET. Early work by [11] and Tiepolt et al. (2016) demonstrated that early-phase [^11^C]PiB and [^1^⁸F]florbetaben uptake closely parallels FDG metabolism and correlates with cognitive performance, indicating that early-phase amyloid PET captures neuronal injury rather than amyloid binding. Later studies confirmed these findings across different tracers and acquisition protocols, showing strong correspondence between early-phase perfusion and metabolic patterns, as well as sensitivity to cognitive impairment [5, 6, 14]. However, longitudinal evidence has been scarce; most prior studies focused on cross-sectional associations, limiting inference about prognostic value. The present results fill this gap by demonstrating that baseline perfusion predicts the rate of cognitive decline over several years, complementing molecular amyloid information obtained from the late phase.

These findings are also consistent with FDG-PET literature showing that hypometabolism in AD-vulnerable regions predicts cognitive deterioration [24, 27]. The convergence between FDG- and early-phase amyloid PET results supports the use of dual-phase amyloid imaging as a practical and informative approach for assessing both molecular and functional aspects of neurodegeneration in a single session.

These findings also align with literature showing substantial regional overlap between tau deposition and functional deficits measured by FDG- or early-phase amyloid PET, indicating that perfusion reflects downstream neuronal dysfunction.

### Clinical and research implications

Our findings support the use of early-phase amyloid PET perfusion as a complementary biomarker that captures functional aspects of neurodegeneration without the need for additional metabolic imaging such as FDG-PET. This is particularly relevant in clinical practice, where diagnostic efficiency, cost, and patient burden are critical considerations.

From a clinical standpoint, early-phase perfusion in AD-related regions may provide prognostic information within amyloid-positive individuals—those most likely to follow the Alzheimer’s disease trajectory and to be considered for disease-modifying therapies. In this population, lower baseline perfusion was associated with faster cognitive decline, indicating that perfusion reflects functional integrity beyond amyloid burden alone. In contrast, the absence of significant effects in amyloid-negative participants likely reflects the use of an AD-specific meta-ROI rather than a true lack of a perfusion–cognition relationship. Broader regional analyses may reveal distinct perfusion patterns relevant to non-AD pathologies.

From a research perspective, incorporating early-phase perfusion measures into longitudinal and interventional studies could improve sensitivity to dynamic neuronal and vascular changes that accompany clinical decline. This single-visit dual-phase PET approach—combining molecular and functional readouts acquired in the same session—may enhance understanding of disease mechanisms and improve the design and interpretation of therapeutic trials by providing a more comprehensive view of neurodegenerative processes over time.

### Limitations

Several limitations should be noted. Although all participants had longitudinal data, missing follow-up visits may have reduced power to detect subtle cognitive change. PET imaging was performed with two tracers and across multiple scanners, which could introduce variability; however, control analyses indicated that tracer type did not significantly affect perfusion values or group differences (see Methods). Structural MRI was available for all participants, but FLAIR-based ratings of white matter lesions (ARWMC) and APOE ε4 genotype were obtained only in subsets, limiting sample size for covariate-adjusted models. Because the study sample was drawn from a single memory-clinic cohort, external validation across multiple centers and populations will be important to confirm the generalizability of the findings.

Cognitive change was assessed exclusively with the Mini-Mental State Examination (MMSE). While the MMSE remains the most widely used brief cognitive screening tool, it lacks sensitivity to subtle or domain-specific decline—particularly in early or atypical disease stages [28–30]. Nevertheless, the MMSE continues to play a central role in both clinical research and regulatory frameworks for Alzheimer’s disease, as current treatment recommendations for anti-amyloid monoclonal antibodies specify eligibility for biomarker-confirmed amyloid positivity together with mild cognitive impairment or mild dementia typically defined by MMSE scores between 22 and 30 [31, 32]. Thus, while the MMSE is clinically relevant, future studies should combine it with more comprehensive and domain-specific neuropsychological measures or composite cognitive scores to capture subtle longitudinal changes and to refine the mapping between perfusion and cognitive function.

The meta-ROI used for perfusion quantification was derived from regions typically affected in Alzheimer’s disease. This approach improves comparability with prior work but may not capture region-specific perfusion changes in atypical AD variants or non-AD dementias. Exploring alternative ROI definitions or voxel-wise approaches could help delineate spatially heterogeneous or non-canonical perfusion patterns across diagnostic subtypes.

Finally, tau-PET data were not available, precluding direct tests of perfusion–tau coupling. Future studies combining perfusion and tau imaging will help clarify whether reduced perfusion reflects downstream neuronal dysfunction linked to regional tau pathology.

Despite these limitations, the present study provides novel evidence that early-phase amyloid PET–derived perfusion is linked to longitudinal cognitive change, thereby laying the groundwork for future multimodal and prognostic investigations.

### Future directions

Future studies should move beyond global composite ROIs to investigate region-specific perfusion patterns across clinical phenotypes. Such analyses may reveal more precise functional signatures of disease progression or resilience and better align perfusion markers with domain-specific cognitive changes. Incorporating broader neuropsychological batteries and composite cognitive scores will be essential to capture subtle changes that cannot be detected with brief global measures such as the MMSE.

Further work should also examine how early-phase perfusion relates to regional amyloid deposition and other molecular markers. In the present study, perfusion showed only a modest inverse correlation with global Centiloid values, suggesting partially independent contributions of vascular and amyloid pathology. A voxel-wise comparison of late-phase amyloid SUVr maps between amyloid-positive individuals with low versus high perfusion could clarify whether regional amyloid distribution modulates perfusion deficits. Such analyses, combined with region-specific or network-based ROI approaches, may help disentangle the interplay between amyloid burden, vascular compromise, and neuronal dysfunction.

Integrating tau-PET, fluid biomarkers, and longitudinal perfusion in larger prospective cohorts will be essential to validate perfusion as a dynamic, tau-aligned marker of neurodegeneration.

## Conclusion

Perfusion derived from early-phase amyloid PET was associated with baseline cognitive performance and predicted longitudinal cognitive decline among amyloid-positive individuals. These findings indicate that early-phase perfusion reflects downstream neuronal dysfunction related to Alzheimer’s pathology and provides prognostic information beyond amyloid burden alone. By capturing both molecular and functional aspects within a single dual-phase acquisition, this approach enhances diagnostic and prognostic efficiency without the need for additional metabolic imaging. Clinically, early-phase perfusion may support patient stratification and monitoring in the context of disease-modifying treatments.

## Supplementary Information

Below is the link to the electronic supplementary material.

Supplementary file1 (DOCX 3327 KB)

## Data Availability

The datasets generated during and/or analysed during the current study are available from the corresponding author on reasonable request.
