# Supplementary material for: Hypoperfusion in early-phase amyloid PET as a predictor of cognitive decline in Alzheimer's disease
Source: Eur J Nucl Med Mol Imaging. 2026 Mar 2;53(6):4037–49. doi: 10.1007/s00259-025-07694-1 (PMC13121204; doi:10.1007/s00259-025-07694-1)
Supplement: Supplementary file 1 — Supplementary file1 (DOCX 3327 KB) [file 259_2025_7694_MOESM1_ESM.docx]

## 1. MRI acquisition

High-resolution anatomical 3D T1-weighted MRI scans were acquired on two different 3T scanners, depending on scanner availability: the MR750w (GE Healthcare, Milwaukee, WI) and the Magnetom Skyra (Siemens Healthineers, Erlangen, Germany). The primary acquisition protocol on the GE system used the following parameters: repetition time (TR) = 1930 ms, echo time (TE) = 2.36 ms, flip angle = 8°, field of view = 256 × 256 mm, matrix size = 288 × 288, and slice thickness = 0.9 mm. An alternative protocol was used on the Siemens scanner, with matrix size = 254 × 254, 178 slices, isotropic voxel resolution of 1 mm³, and TR = 7.2 ms. Both protocols yielded high-resolution structural images suitable for volumetric analysis and PET–MRI co-registration.

## 2. PET acquisition

All scanners were from the same vendor and of the same generation, harmonized regarding their performance and reconstructions, and cross-calibrated. Amyloid-PET: [¹⁸F]Florbetapir (¹⁸F-FBP) late-phase images were acquired 50 minutes after the intravenous administration of 210 ± 18.77 MBq (3 × 5-minute image frames). [¹⁸F]Flutemetamol (¹⁸F-FMM) late-phase images were acquired 90 minutes after the intravenous administration of 166 ± 16.73 MBq (4 × 5-minute image frames). Images were then averaged into a single 15- or 20-minutes frame. All amyloid-PET late images were visually assessed by an independent, board-certified specialist in nuclear medicine (VG) applying the standard operating procedures approved by the European ([Amyvid, INN-florbetapir [18F]](https://www.ema.europa.eu/documents/product-information/amyvid-epar-product-information_en.pdf)). Subjects were classified based on the visual inspection of the late images into "Aβ+” or "Aβ-” (i.e., subjects that presented high levels or low levels of cortical amyloid binding, respectively). Data were acquired in list mode and were reconstructed using 3D OSEM 4 interactions 8 subsets, and a 2 mm Gaussian filter at FWHM, resulting in images with a 400 x 400 matrix with 1.01 mm isotropic voxels.

## 3. MRI and PET normalization processing

MRI 3D T1-weighted sequences were aligned to a reference plane passing through the anterior and posterior commissures, segmented into gray matter, white matter, and cerebrospinal fluid compartments, and normalized to the Montreal Neurologic Institute (MNI) space using tissue probability maps.

Early-phase eFBP and FMM PET images were co-registered to the individual T1 MRI and normalized to the MNI space using the transformation matrix obtained during MRI normalization. All PET images were spatially smoothed with an isotropic 3D Gaussian kernel (8 mm FWHM).

Mean standardized uptake values (SUV) were extracted from predefined regions-of-interest using the AAL3 atlas. The target meta-ROI was defined according to Landau et al. (2011) and included the bilateral angular gyri, posterior cingulate cortex, and left middle/inferior temporal gyrus. The reference region consisted of the pons and cerebellar vermis, following the ADNI implementation by Landau et al. (2011). SUVr values were computed as volume-weighted mean target-to-reference ratios, where voxelwise SUV values were averaged within each ROI, accounting for regional size differences.

####

#### Table S1

Demographic and clinical characteristics of participants with available APOE genotype data (n = 140).

#### Table S2
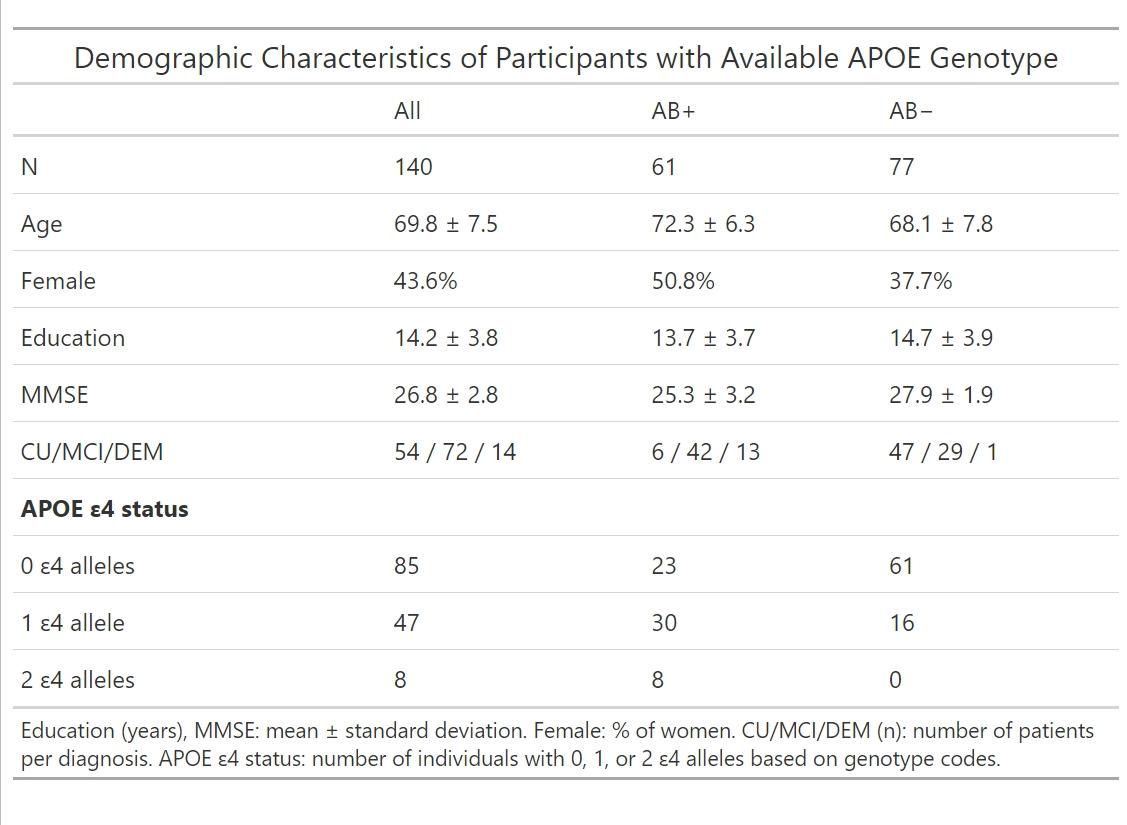


Demographic and clinical characteristics of participants with available ARWMC data (n = 155).


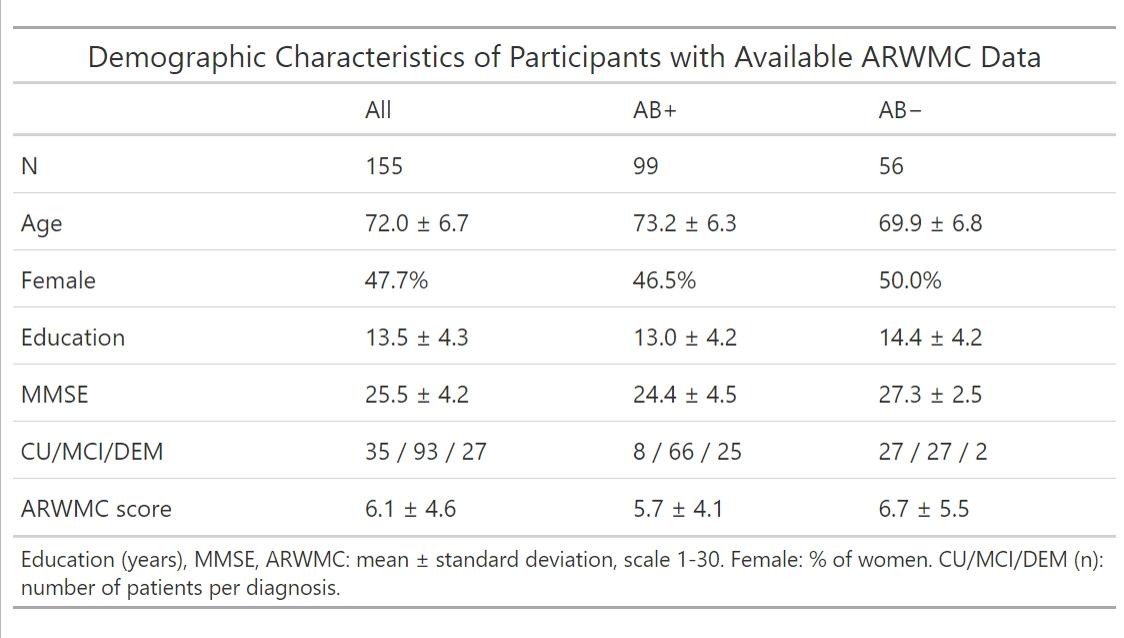


## 4. Statistical analysis

### 4.1. Handling of missing data

Table S3

Summary of missing data patterns and logistic regression analyses for APOE and ARWMC.


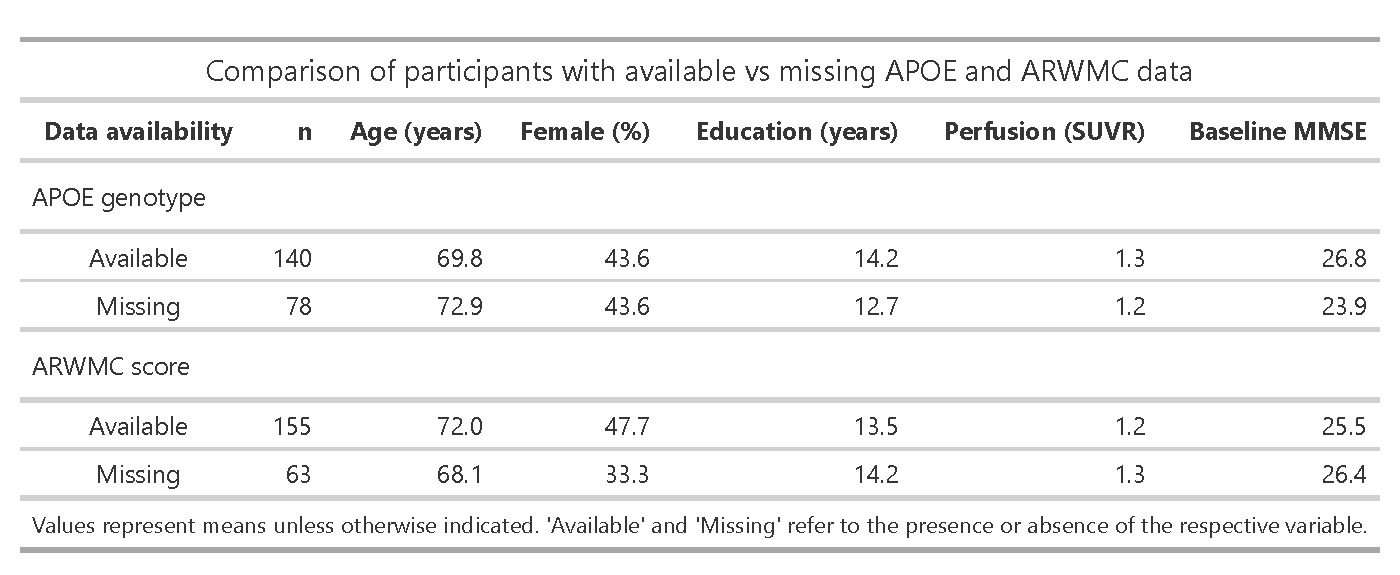


### 4.2. Cross-sectional analyses

#### Table S4

Group differences in early-phase perfusion (meta-ROI SUVr). Mean early-phase PET perfusion values (SUVr ± SD) are shown for cognitively unimpaired (CU), mild cognitive impairment (MCI), and dementia (DEM) participants in the full cohort and stratified by amyloid status. Superscripts denote Tukey’s HSD post-hoc groups (values sharing the same letter do not differ, p < 0.05). Aβ subgroups exclude borderline visual reads.Mean early-phase PET perfusion values (SUVr ± SD) are shown for cognitively unimpaired (CU), mild cognitive impairment (MCI), and dementia (DEM) participants in the full cohort and stratified by amyloid status. Superscripts denote Tukey’s HSD post-hoc groups (values sharing the same letter do not differ, p < 0.05). Aβ subgroups exclude borderline visual reads.

###
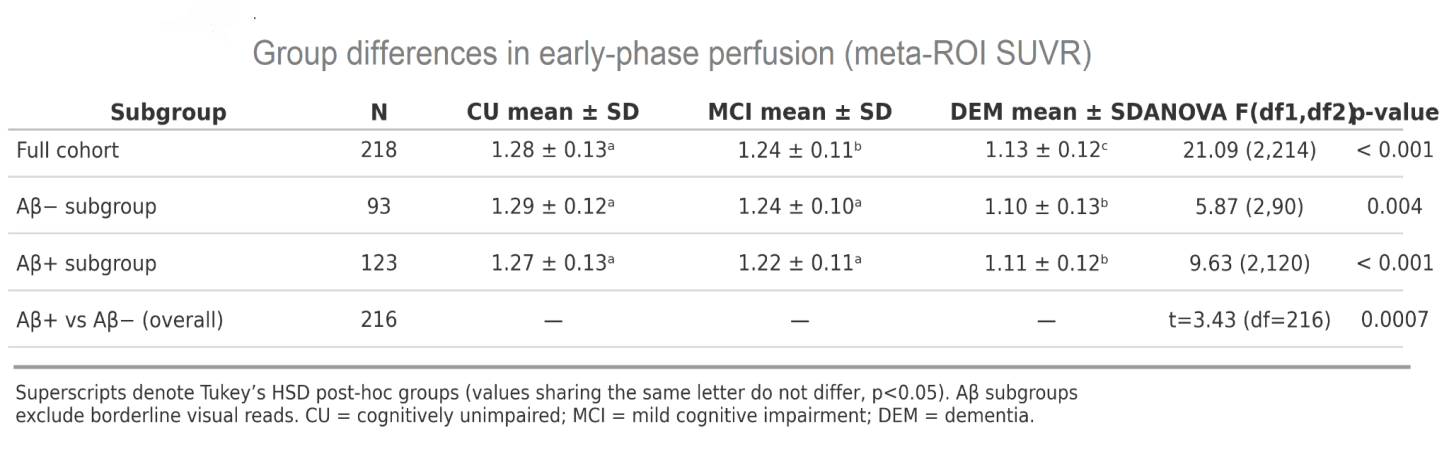


### 4.3. Longitudinal analysis (LME models)

Linear mixed-effects models 1a–3 (full cohort and stratified subgroups): fixed-effects estimates (β, SE, 95% CI, p). All effects remained significant after Holm–Bonferroni correction (see Supplementary Table 6 for adjusted p-values).

Table S5


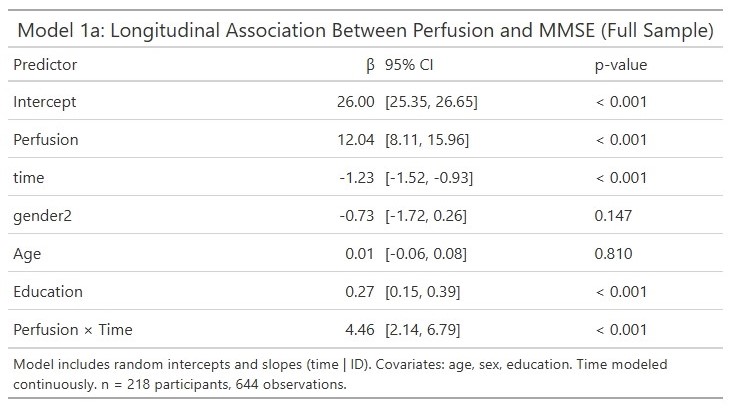


Table S6


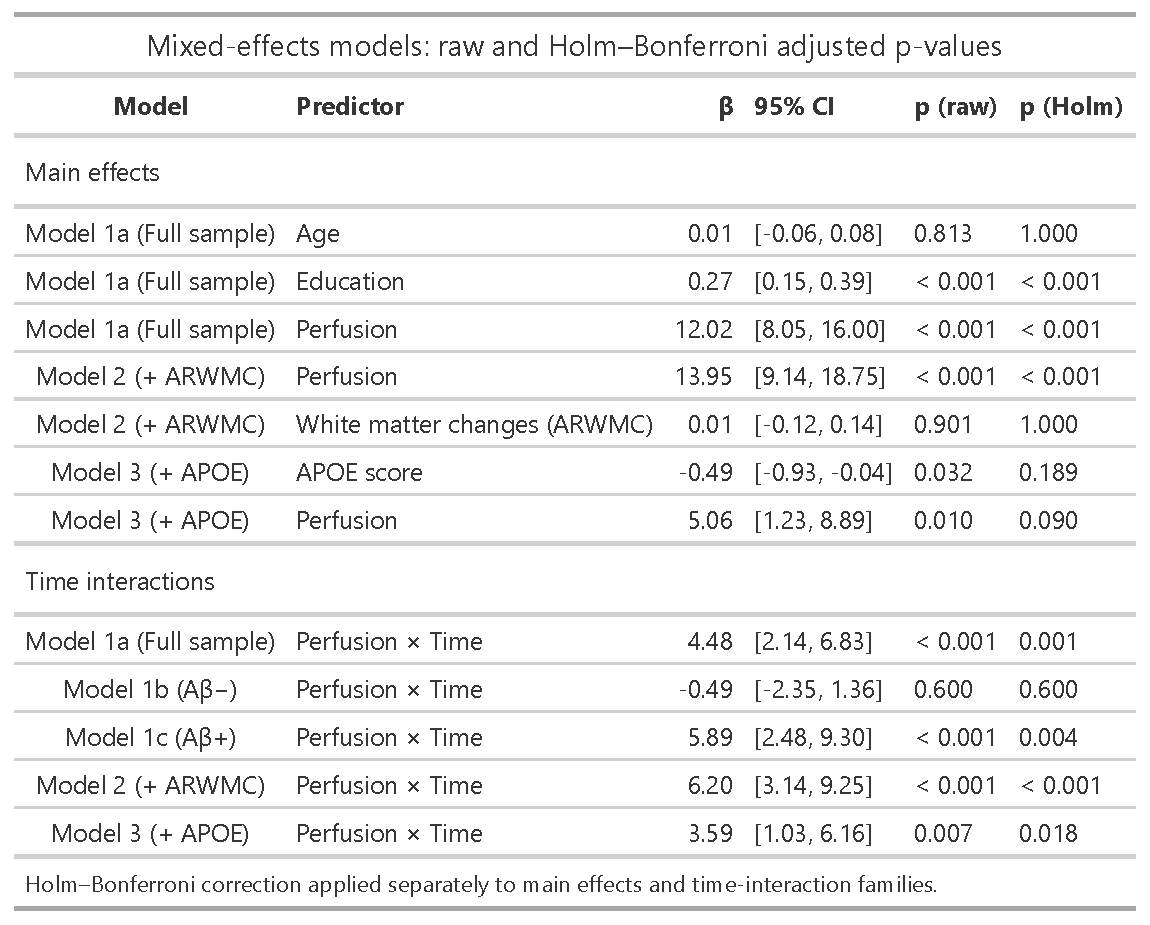


Table S7


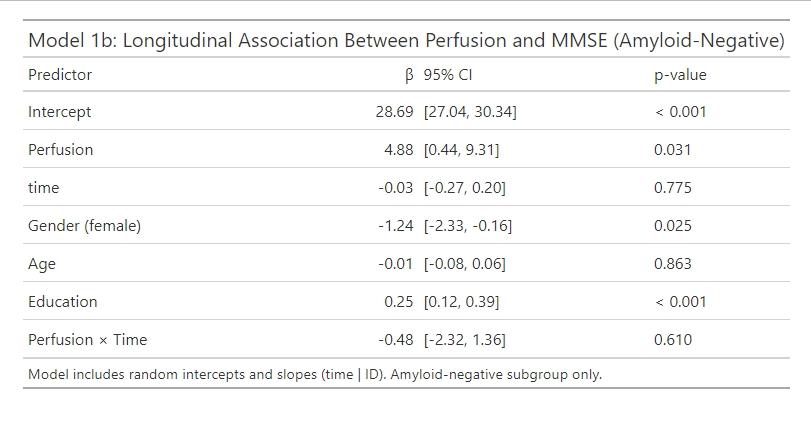


Table S8


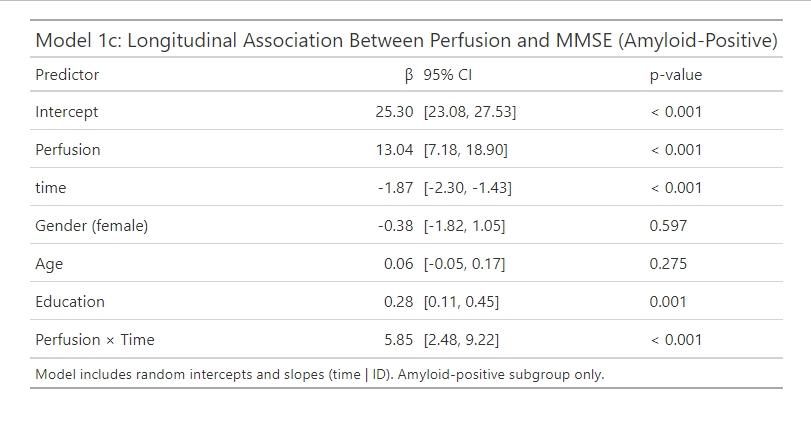


Table S9


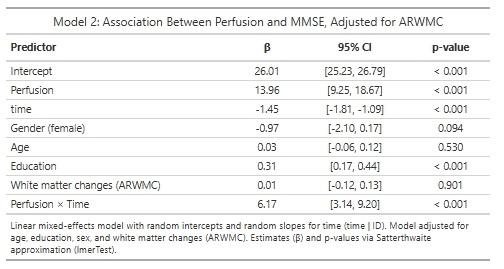


Table S10


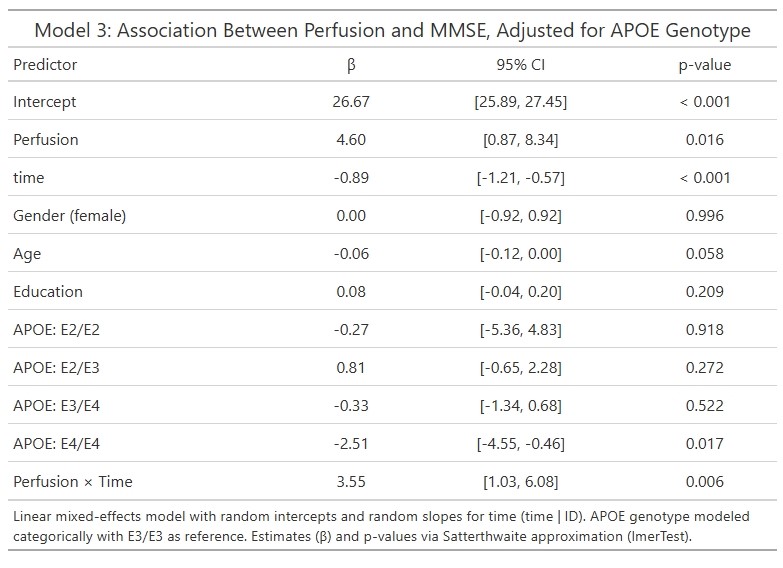


Table S11


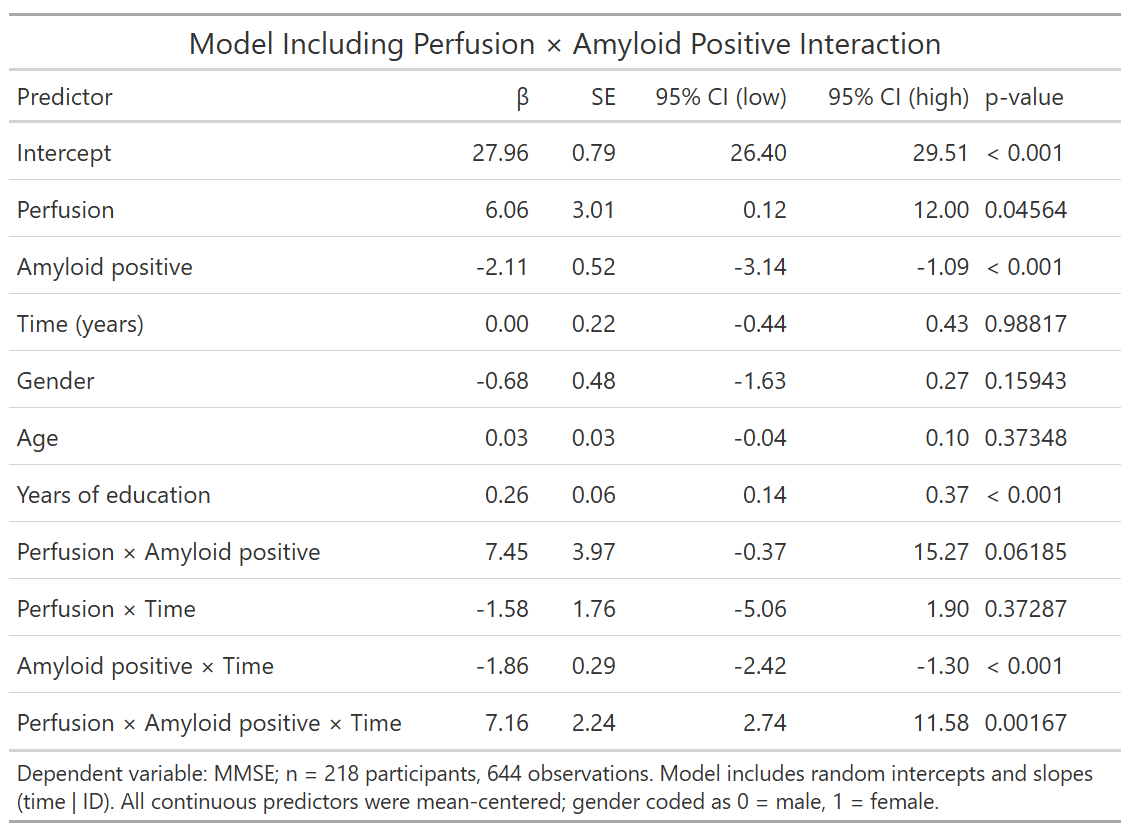


## 5. Extended Analyses: Perfusion vs. Amyloid (Centiloid)

#### Table S12

Linear mixed-effects model using Centiloid as predictor (analog to Model 1a).


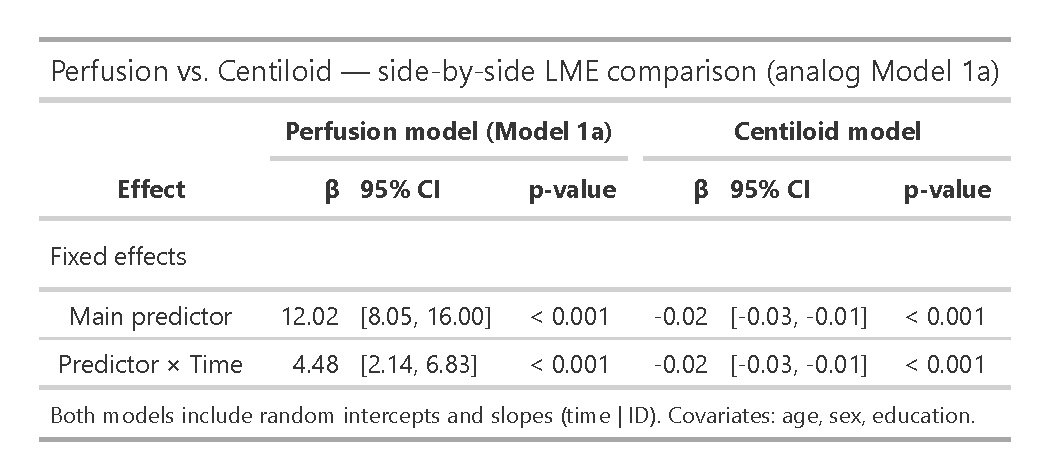


####

#### Table S13

Comparison of perfusion and Centiloid models (AIC/BIC fit indices).


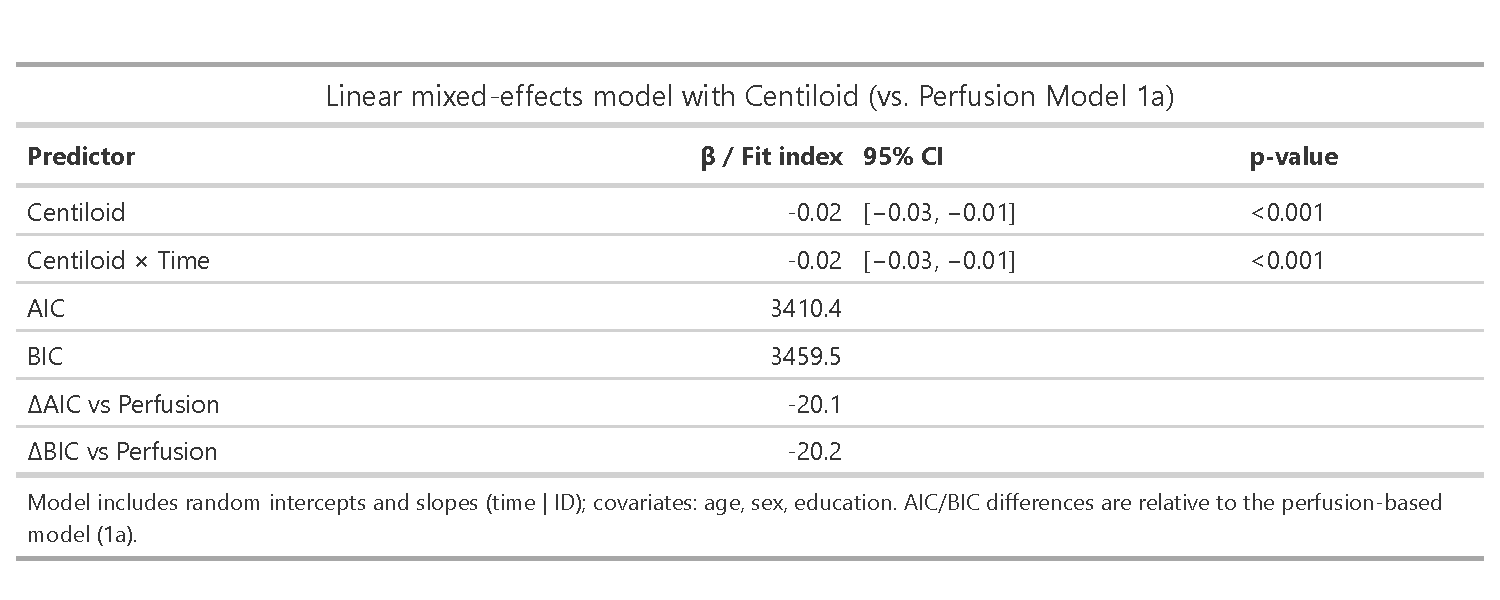


#### Table S14

Spearman correlation (ρ = –0.26) and ROC analysis (AUC = 0.68) between perfusion SUVr and Centiloid.
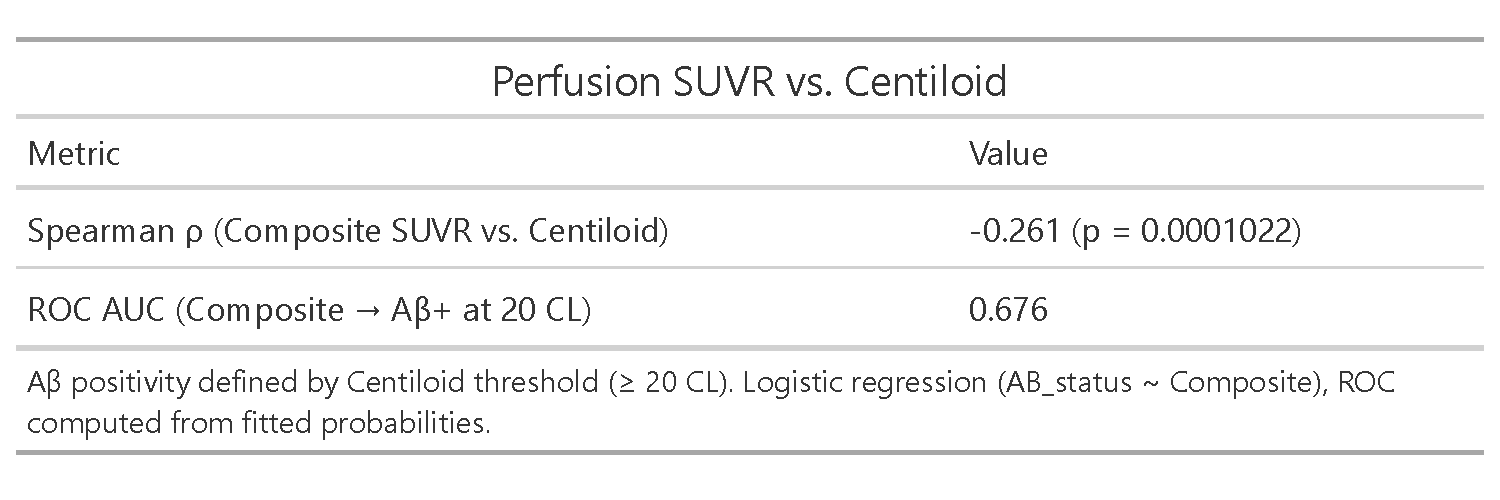


#### Table S15

Pearson and Spearman correlations and linear regression results examining the relationship between global amyloid burden (Centiloid) and early-phase perfusion (Composite SUVr). These results correspond to the scatter plot shown in Supplementary Figure S1.


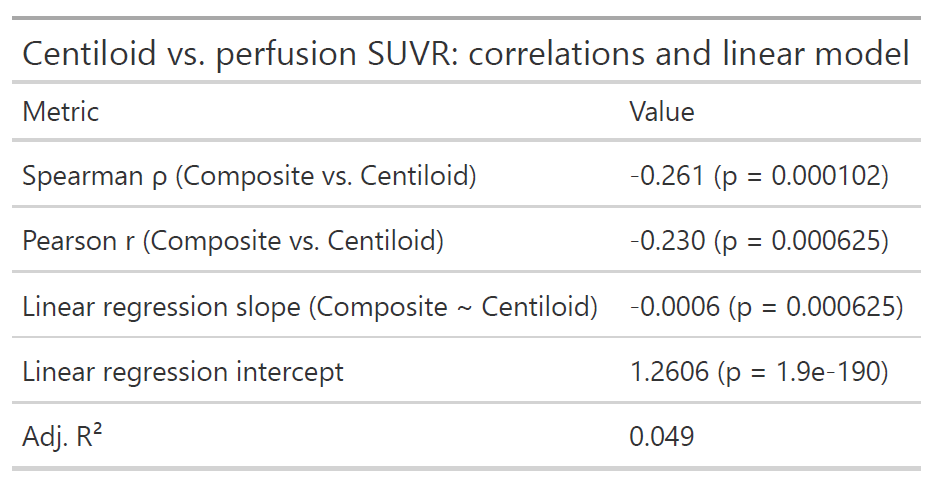


#### Figure 1

Each point represents one participant. The red line indicates the linear regression fit (95% CI shaded). The vertical dashed line marks the Centiloid threshold of 20 used to define amyloid positivity (Aβ+). Spearman ρ = –0.26, p = 0.0001; Pearson r = –0.23, p = 0.0006.


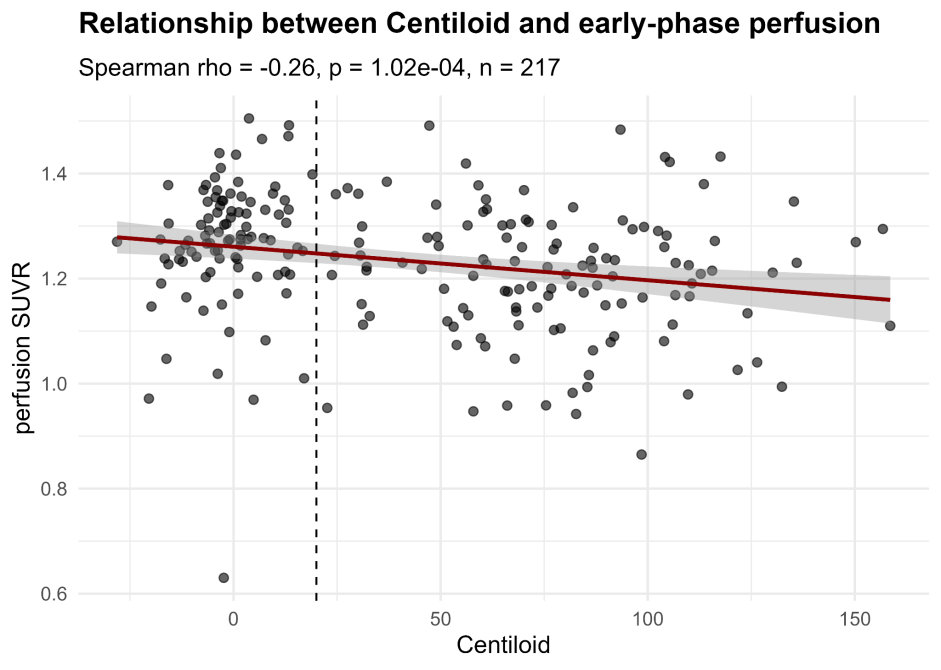


#### Table S16

Exploratory mixed-effects model assessing the joint and interactive effects of early-phase perfusion (Composite SUVr) and amyloid burden (Centiloid, continuous variable) on longitudinal MMSE performance. The model included fixed effects for perfusion, Centiloid, time, their two-way and three-way interactions, and covariates age, sex, and years of education. Random intercepts and slopes were specified for time (time | ID).


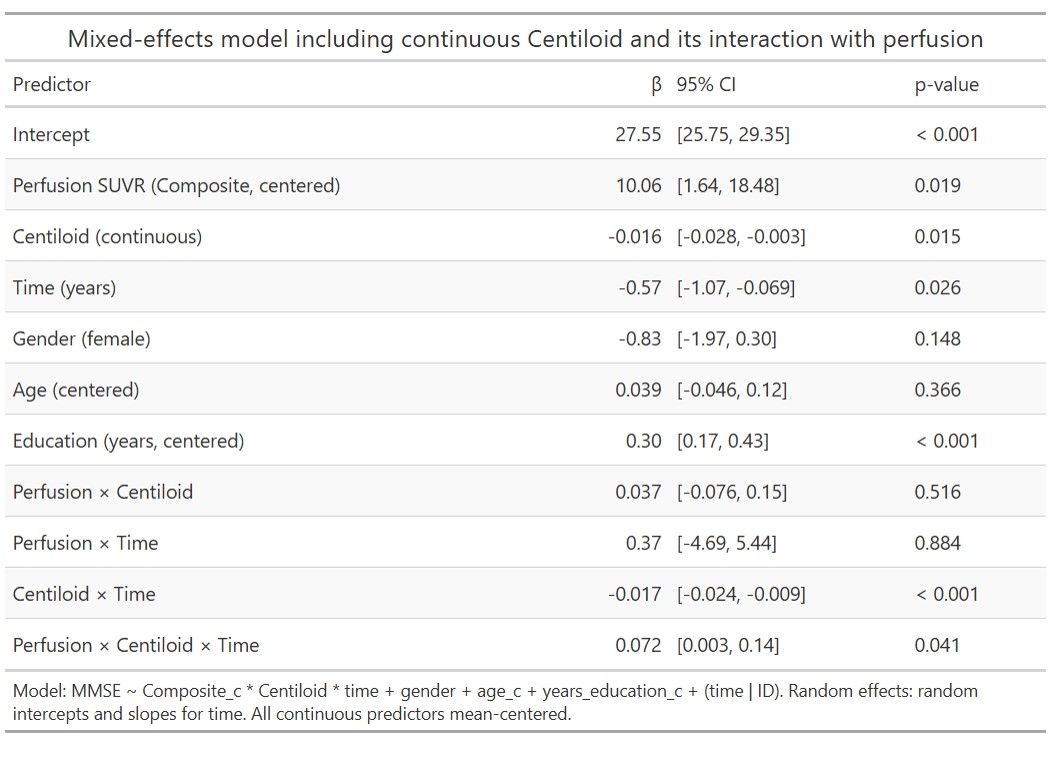


#### Table S17


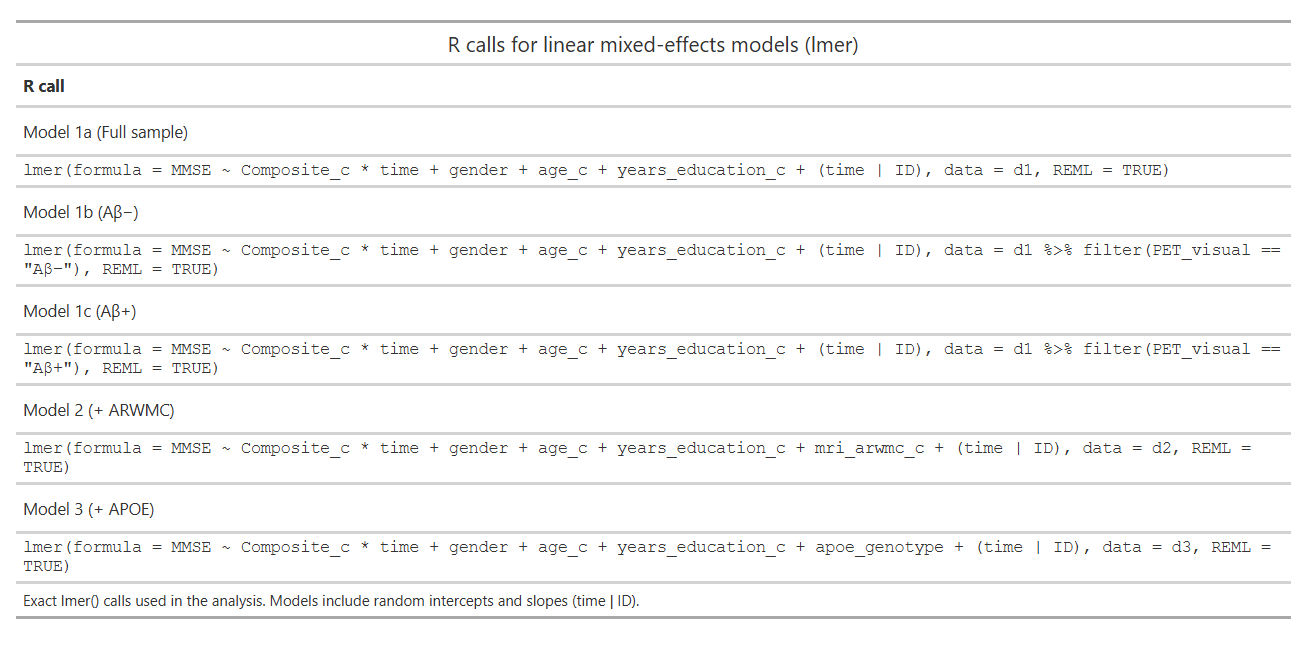
Exact R syntax used to fit linear mixed-effects models (Models 1–3).

## 6. Sensitivity Analysis by Radiotracer Type
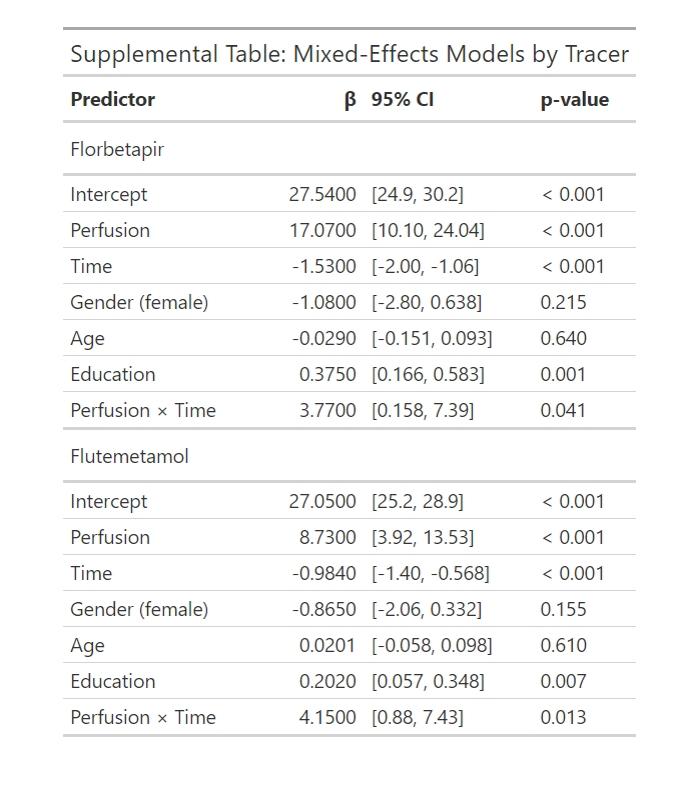


To account for potential variability introduced by different radiotracers, we repeated the primary longitudinal analysis (Model 1a) separately for participants scanned with Florbetapir and Flutemetamol. The results were broadly consistent across tracers, though effect sizes varied slightly (see Table S4).

##

## 
